# Virtually delivered Mindfulness-Oriented Recovery Enhancement (MORE) reduces daily pain intensity in patients with lumbosacral radiculopathy: a randomized controlled trial

**DOI:** 10.1097/PR9.0000000000001132

**Published:** 2024-03-14

**Authors:** Ryan S. Wexler, Devon J. Fox, Danielle ZuZero, Melissa Bollen, Anand Parikshak, Hannah Edmond, Johnny Lemau, Diane Montenegro, Jillian Ramirez, Sophia Kwin, Austin R. Thompson, Hans L. Carlson, Lynn M. Marshall, Thomas Kern, Scott D. Mist, Ryan Bradley, Douglas A. Hanes, Heather Zwickey, Courtney K. Pickworth

**Affiliations:** a Helfgott Research Institute, National University of Natural Medicine, Portland, OR, USA; b Center for Research and Training, Department of Wellness and Preventive Medicine, Cleveland Clinic, Cleveland, OH, USA; c Department of Orthopaedics and Rehabilitation, Oregon Health & Science University, Portland, OR, USA; d Department of Anesthesiology and Perioperative Medicine, Oregon Health & Science University, Portland, OR, USA; e Herbert Wertheim School of Public Health and Human Longevity Sciences, University of California, San Diego, La Jolla, CA, USA

**Keywords:** Lumbosacral radiculopathy, Mindfulness-Oriented Recovery Enhancement, Chronic low back pain, Mindfulness-based interventions, Integrative pain management

## Abstract

**Introduction:**

Lumbosacral radiculopathy (LR), also known as sciatica, is a common type of radiating neurologic pain involving burning, tingling, and numbness in the lower extremities. It has an estimated lifetime prevalence as high as 43%.

**Objectives:**

The objective of this randomized controlled trial was to evaluate the impact of virtually delivered Mindfulness-Oriented Recovery Enhancement (MORE) on patients with LR during the COVID-19 pandemic.

**Methods:**

Potentially eligible patients were identified using electronic health record queries and phone screenings. Participants were then randomized to MORE or treatment-as-usual (TAU) for 8 weeks, with pain intensity assessed daily. At baseline and follow-up visits, participants completed questionnaires assessing the primary outcome, disability, as well as quality of life, depression, mindful reinterpretation of pain, and trait mindfulness.

**Results:**

In our study, patients undergoing virtual delivery of MORE had greater improvements in daily pain intensity (*P* = 0.002) but not in disability (*P* = 0.09), depression (*P* = 0.26), or quality of life (*P* = 0.99 and *P* = 0.89, SF-12 physical and mental component scores, respectively), relative to TAU patients. In addition, patients in MORE experienced significantly greater increases in mindful reinterpretation of pain (*P* = 0.029) and trait mindfulness (*P* = 0.035).

**Conclusion:**

Among patients with lumbar radiculopathy, MORE significantly reduced daily pain intensity but did not decrease disability or depression symptoms. Given the long duration of symptoms in our sample, we hypothesize the discrepancy between changes in daily pain intensity and disability is due to fear avoidance behaviors common in patients with chronic pain. As the first trial of a mindfulness intervention in patients with LR, these findings should inform future integrative approaches to LR treatment, particularly when considering the increasing use of virtual interventions throughout the COVID-19 pandemic.

## Abstract 2

Supplemental Digital Content is Available in the Text.

## 1. Introduction

In 2020, the rate of persistent chronic pain was 462 per 1000 person-years, making it a highly prevalent public health concern.^56^ Lumbosacral radiculopathy (LR), sometimes referred to as sciatica or lumbar radiating pain, is a specific type of chronic neurologic pain that involves radiating sensations of pain, burning, tingling, or numbness down 1 or both legs. Many cases of LR will evolve to mixed pain (nociceptive, neuropathic, and nociplastic),^23^ which can last for years or decades as a result of central sensitization.^57^ Given the potential for chronicity and the high lifetime prevalence of LR, as high as 43%,^46^ mitigating the development of nociplastic pain and managing risk factors such as fear avoidance,^42^ sedentary behaviors,^14,36^ and social isolation^8,44,48^ is crucial for patients—especially those with low health-related self-efficacy and worsening disability accentuated during the COVID-19 pandemic.^11^

Because of risks associated with long-term pharmaceutical pain management, organizations such as the National Institutes of Health HEAL Initiative and the International Association for the Study of Pain have shifted their focus towards integrative and nonpharmacologic management of chronic pain—decisions informed in part by decades of work on the application of mindfulness-based interventions (MBI).^1,34,76^ The American College of Physicians already recommends nonpharmacologic treatments such as chiropractic manipulations,^72^ tai-chi, yoga, and mindfulness-based stress reduction as first-line treatment for chronic low back pain (cLBP).^65^ In addition to LBP,^5,12,35^ MBIs have been studied for various neuropathic conditions including fibromyalgia,^3^ diabetic neuropathy,^39^ and chemotherapy-induced peripheral neuropathy.^70^

Mindfulness-Oriented Recovery Enhancement (MORE) is a mindfulness-based intervention that is designed as a therapeutic tool for disrupting the bidirectional and self-perpetuating relationship between chronic pain and stress. Mindfulness-Oriented Recovery Enhancement integrates training in mindfulness skills to enhance attentional control over pain, attentional bias, and facilitate a shift from affective to sensory processing of pain; reappraisal skills to decrease catastrophizing and facilitate negative emotion regulation; and savoring skills to enhance positive emotional regulation and amplify reward processing.^26,61^ Using savoring, MORE targets dysregulated hedonic patterns. To accomplish this, MORE therapists guide participants through a process of phenomenologic self-discovery and external processing that promotes pain reappraisal and encourages social–observational learning.^29^ Through external processing of meditation experiences through group discussion, participants are encouraged to engage socially, therefore increasing their engagement in mindfulness practice—a process that may augment both quantitative and qualitative outcomes.^50,53^ In a previous randomized clinical trial by Garland et al.,^26^ MORE was compared with supportive group therapy for patients with chronic pain and a history of opioid misuse. This design allowed for the control of group factors such as social support and development of therapeutic relationships. The authors found that MORE reduced the occurrence of opioid misuse by 45% at 9-month follow-up, more than doubling the effect of supportive group therapy. In addition, MORE was superior in reducing pain-related functional interference and emotional distress.

Despite the growing body of evidence on MBIs, it remains a small area of interest that is not included in evidence maps and systematic reviews on psychological interventions for chronic pain.^9,37,51^ This article shares findings from self-report questionnaires in the trial described by Wexler et al.,^75^ which were collected during the peak of the COVID-19 pandemic. The primary aims of this research are to evaluate the impact of MORE on disability, pain, quality of life, depression, mindful reinterpretation of pain, and trait mindfulness scores as compared to treatment-as-usual (TAU) in patients with LR. We hypothesized that participants undergoing training in MORE would experience improvements relative to TAU participants in disability, pain, quality of life, depression, mindful reinterpretation of pain, and trait mindfulness.

## 2. Methods

This study adheres to Consolidated Standards of Reporting Trials (CONSORT) guidelines, is registered at clinicaltrials.gov (NCT04818606), and was approved by the National University of Natural Medicine (NUNM) IRB (IRB#: KP112720). Data were collected following the previously described protocol by Wexler et al. (2022) with only minor protocol modifications; these changes are described in detail below.^75^

### 2.1. Recruitment and participants

In summary, adults with LR were randomized to MORE or TAU for 8 weeks. Patients were eligible if meeting the following criteria: presence of radiculopathy symptoms extending below the knee secondary to LBP for greater than 6 weeks with a painDETECT score greater than 18 or previous diagnosis of LR (*ICD-10* M54.16, M54.17, M51.16, M51.17, M47.26, M47.27, M54.40, M54.41, M54.42, M99.53, M99.54, S34.21, S34.22, G54.4, and G55); 18 to 65 years of age; ability to read and understand English; willingness to be randomized to either group; willingness to refrain from self-directed treatment plan changes; daily access to the internet; have not received an epidural steroid injection of LR in the previous 3 months; have not received a surgical intervention for LR in the previous 6 months; ability to complete 20 unassisted gait cycles; does not have a regular mindfulness practice of at least once a week; does not have a diagnosis of cancer; does not have an allergy to adhesive; and does not have an unmanaged or uncontrolled mental illness known to cause psychosis.

Most eligible participants were identified and contacted through electronic medical records systems queries within the NUNM Health Center, the Oregon Health & Science University Spine Center, and the Oregon Health & Science University Comprehensive Pain Center. Electronic health records queries were conducted using the aforementioned eligibility criteria as filters. Patients were initially contacted through email and followed up through phone 1, 2, and 4 weeks thereafter. A final email was sent to all patients not reached through phone before closing recruitment. In total, this search strategy included patients under the care of providers from at least 8 health care specialties, including acupuncture, chiropractic, interventional radiology, massage, naturopathy, nurse practitioner, psychology, and psychiatry.

### 2.2. Study visits

All baseline study visits were conducted in-person at Helfgott Research Institute at NUNM in Portland, Oregon. Baseline visits included a review of participant eligibility, a discussion and signing of the informed consent, completion of self-report questionnaires, and surface electromyography testing (to be reported elsewhere). Baseline study visits lasted between 30 and 75 minutes, depending on participant questions and timeliness in survey completion. Follow-up visits contained, at most, the self-report questionnaires and surface electromyography testing; because of the nature of the ongoing COVID-19 pandemic at the time of study implementation, participants were offered the opportunity to complete follow-up visits virtually, with self-report questionnaires delivered remotely through Research Electronic Data Capture (REDCap). Study data were collected and managed using REDCap electronic data capture tools hosted at NUNM.^31,32,47^ REDCap is a secure, web-based software platform designed to support data capture for research studies, providing (1) an intuitive interface for validated data capture; (2) audit trails for tracking data manipulation and export procedures; (3) automated export procedures for seamless data downloads to common statistical packages; and (4) procedures for data integration and interoperability with external sources.

### 2.3. Interventions

#### 2.3.1. Treatment-as-usual

Participants in the control group, TAU, were asked to maintain their current treatment regimen, including but not limited to physical therapy, oral anti-inflammatories, acupuncture, chiropractic, massage, etc., and asked to report treatment plan changes made while enrolled in the study. Treatment plan changes were made reportable through a daily survey containing a visual analogue scale (VAS) for pain intensity that was delivered by text using Twilio^16^ or email, dependent on participant preference. Altogether, TAU group participants received a daily survey containing 3 questions: (1) the daily VAS, (2) any treatment plan changes made, and (3) if treatment plan changes were made, please describe them. Treatment-as-usual group participants were added to a waitlist to participate in the MORE program after cessation of data collection, as is conventional in mindfulness studies.

#### 2.3.2. Mindfulness-Oriented Recovery Enhancement + treatment-as-usual

Mindfulness-Oriented Recovery Enhancement is an 8-week mindfulness intervention with once-weekly 2-hour sessions and regular “homework” throughout the week. Mindfulness-Oriented Recovery Enhancement equips participants with 3 tools or tenets through which to view and manage pain: mindfulness, reappraisal, and savoring.^28^ As noted in session descriptions available in Appendix A, http://links.lww.com/PR9/A223, all 3 of these tools are introduced within the first 4 weeks of the program and then built upon in subsequent sessions. During sessions, participants are guided through mindfulness practices, group discussion, and pain education as manualized (instructor training and qualifications are described in the study protocol).^28^ Participants were sent weekly reminders about synchronous sessions before class each week and followed up through phone, if absent, to troubleshoot technology challenges and barriers to participation. As this study was conducted at the height of the COVID-19 pandemic, MORE was delivered virtually each week. To join the session, participants joined a Zoom meeting using an anonymized screen name, which was self-selected at the participants' baseline visit. Participants were encouraged to keep their video on during class but were provided the option to remain off camera for comfort or anonymity, if preferred.

Participants who were unable to attend sessions were provided with recorded session audio to review on their own, to stay on schedule with educational content. Participants were also provided with recorded guided meditations from the MORE instructor and homework to complete throughout the week. These instructions were provided in regular follow-up emails after the weekly session.

As with TAU, MORE participants were asked to maintain other elements of their treatment plan the same as at baseline and report changes made during enrollment. In addition to the 3 questions asked of TAU participants in the daily survey, MORE participants were also provided a space to share reflections from their practice and asked to report their daily practice time. As described in the study protocol, all study participants received a copy of the pain education handout, “Understanding Pain,” provided publicly by the Oregon Pain Management Commission, at the time of intervention initiation.^60^

### 2.4. Outcome measures and data collection

The present analysis includes all 7 self-report outcome measures used in this study: Modified Oswestry Disability Index (ODI), painDETECT Questionnaire (PDQ), Pain VAS, Major Depressive Inventory (MDI), SF-12 Quality of Life Questionnaire (SF-12), Mindful Reappraisal of Pain Scale (MRPS), and the Five-Facet Mindfulness Questionnaire (FFMQ). Participants completed questionnaires at the baseline and follow-up study visits, whereas the VAS was completed daily through automated REDCap emails or Twilio texts based on participant preference. All study visits were conducted at Helfgott Research Institute or remotely. The ODI was selected as the primary outcome measure for this study because it is frequently used for the assessment of improvement in LR symptoms in studies of steroid injection efficacy—one of the most common treatments for LR.^4,13,20,58,59^ In addition to symptom-specific questionnaires, a demographics and health history questionnaire was designed by the study team to capture information on diagnosis, condition duration, and past and current treatments. With the exception of the MRPS, all questionnaires are described in detail in the original study protocol,^75^ including their rationale for use in this subpopulation.

The MRPS is a self-report questionnaire designed to quantify participant mindfulness practices as it relates to mindful reappraisal of pain.^27^ The MRPS is adapted from the reinterpreting pain sensations subscale of the Coping Strategies Questionnaire^66^ to use language relevant to the teaching content common to MBIs. In addition to questions adapted from the Coping Strategies Questionnaire, additional questions were built into the MRPS specific to meditation practices, such as focusing attention on the breath and changing body sensations. Evaluated in samples of opioid-treated patients with chronic pain, the MRPS is a 9-item survey measured on a scale from 0, “never do that,” to 6, “always do that,” with responses summed to create a composite score. It was found to have good convergent and discriminant validity with various mindfulness and coping questionnaires, to be highly sensitive to change in participants undergoing mindfulness training, and to mediate the effect of MORE on pain severity as measured by the brief pain inventory.^27^

### 2.5. Sample size

Applying estimates of a minimal clinically important difference for the primary outcome measure (ie, the ODI) of 10 points,^17^ an effect size of d = 0.83,^19,24,25^ and *alpha* = 0.05, with power = 80% yielded a required sample size of 48 total participants. At protocol development, an 80% retention rate was assumed, resulting in a necessary recruitment of 60 participants. Sample size calculations are described in detail in the previously published protocol.^75^

### 2.6. Adverse events

Adverse events were evaluated through regular monitoring of daily participant reports and classified as anticipated, unanticipated, and medical emergency. In addition, the mindfulness instructor was informed of the need to communicate adverse events occurring during weekly sessions to the study coordinator and principal investigator. Weekly session audio recordings were reviewed by the study coordinator for potential adverse events. Adverse events occurring during the intervention were logged in a REDCap form and reported to the NUNM IRB. Expected adverse events included mental health exacerbations related to mindfulness practices or to completion of the self-report questionnaires at the study visits. Any adverse events reported directly to the study coordinator or found in weekly audio sessions were subsequently reported to the principal investigator for follow-up with the study participant.

### 2.7. Randomization and blinding

Randomization adhered to the previously published protocol.^75^ Three sequential cohorts of 24 to 46 (12–23 per group) participants were recruited into the study and underwent group assignment through simple randomization using a random number generator. Although this deviates from optimal cohort volume for psychotherapeutic interventions, larger cohorts were necessary because of study resource limitations.^22^ Once maximum enrollment had been reached for a given cohort, randomization was conducted, and subsequent cohort enrollment began. Methods for allocation concealment were previously reported and adhered to.^75^

### 2.8. Statistical analysis plan

Statistical analyses were conducted using SPSS Version 29.0.0.^40^ and adhered to the described protocol.^75^ Briefly, linear mixed modeling with maximum likelihood estimation and random participant intercept was used to assess changes in ODI, MDI, SF-12, MPRS, and FFMQ scores from baseline to follow-up using an intention-to-treat (ITT) design with group × time interaction as the main effect of interest. Potential covariates included age, sex, change in treatment during study enrollment (as a binary variable), disease etiology, duration of symptoms before study enrollment, previous condition-specific surgery, and baseline instrument score. Multilevel modeling with maximum likelihood estimation of missing data was used for growth curve analysis of daily pain VAS data, which has a nested hierarchical structure with both between- and within-subject predictors. The group × time interaction was the primary fixed effect of interest. Models included a random intercept, and the covariance structure for repeated effects (diagonal or AR1) was also evaluated by -2LL fit statistics.

## 3. Results

**Figure 1.**
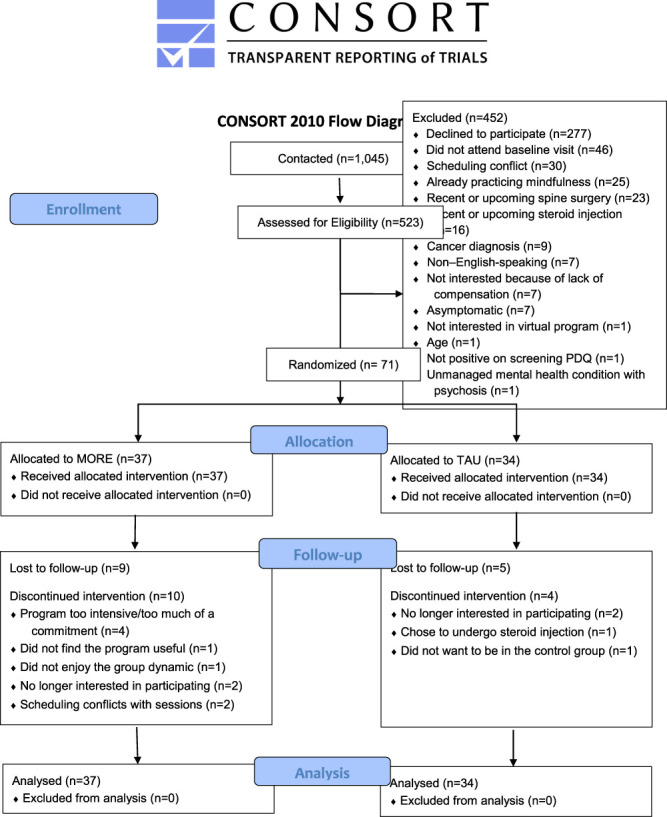
CONSORT diagram. CONSORT, Consolidated Standards of Reporting Trials.

Between January 2021 and January 2022, a team of 8 research staff contacted 139 patients from NUNM and 906 patients from Oregon Health & Science University. Of these 1045 patients contacted, 523 (50%) were assessed for eligibility, and 71 (6.8%) were enrolled in the trial. Dropout and lost to follow-up was 28 (39%). Eleven (15%) patients were recruited from NUNM, 31 (44%) from the SPC, 27 (38%) from the CPC, and 2 (2.8%) from community advertising. Participant dropout and group assignment can be seen in the CONSORT diagram (Fig. 1). Fourteen participants (9 MORE and 5 TAU) were lost to follow-up, and another 14 (10 MORE and 4 TAU) dropped out of the study. Nineteen MORE group participants completed follow-up surveys. Amongst these participants, mean session attendance was 5.28 (±1.99) with 14 participants (74%) receiving the minimal intervention dose. Upon randomization, no significant differences were found between the MORE and TAU groups regarding age, condition duration, sex, race, and scores on any self-report outcome measures. Baseline demographic characteristics, prevalence of disease etiology, and number of patients with common LR symptoms can be seen by group in Table 1 with group comparisons for each outcome measure in Table 2. In addition, because of the high dropout rate in this study, a separate assessment of baseline characteristics was conducted on participants who completed the trial (ie, attended a follow-up visit), vs those who did not (Table 3).

**Table 1:** Demographic characteristics at baseline presented as mean (SD) or n (%).

	MORE (n = 37)	TAU (n = 34)	
Demographics			
Sex			*P* = 0.313*
Male	11 (30%)	14 (41%)	
Female	26 (70%)	20 (59%)	
Race			*P* = 0.219*
Anglo-American	30	25	
Black	2	—	
Asian	—	2	
Hispanic/Latino	2	2	
Middle Eastern	1	1	
More than 1 race	1	4	
Other	1	—	
Age, mean (SD)	48.59 (±11.59)	44.94 (±11.47)	*P* = 0.187
Previous back surgeries			*P* = 0.368*
One surgery	5 (14%)	4 (12%)	
Two surgeries	1 (3%)	—	
Three or more surgeries	2 (5%)	—	
Condition duration (yr), mean (SD)	13.72 (±20.28)	12.76 (±11.28)	*P* = 0.718
Etiology			
L3-L4 disc herniation	1	—	
L4-L5 disc herniation	4	2	
L5-S1 disc herniation	—	1	
Disc herniation of unknown level	1	2	
Osteoarthritis	1	—	
Bone spur	1	—	
Degenerative disc disease	4	4	
Spondylolisthesis	2	0	
Vertebral fracture	1	2	
Inflammation	2	—	
Scoliosis	1	—	
Congenital	1	—	
Central canal stenosis	1	2	
Failed back surgery syndrome	1	1	
Etiology unknown	17	19	
Visual analogue scale for pain intensity	5.14 (±1.78)	5.00 (±2.06)	*P =* 0.770
painDETECT Questionnaire (PDQ)			*P* = 0.170*
PDQ > 18	18 (48.64%)	22 (64.70%)	
PDQ > 12 and ≤18	11 (29.72%)	4 (11.76%)	
PDQ ≤ 12	8 (21.62%)	8 (23.52%)	
Symptoms			
Numbness	27 (73.0%)	25 (73,5%)	
Tingling	27 (73.0%)	24 (70.6%)	
Weakness	24 (64.9%)	23 (67.6%)	
Burning/Electric	27 (73.0%)	26 (76.5%)	

* *P*-value calculated using χ^2^.

MORE, Mindfulness-Oriented Recovery Enhancement; TAU, treatment-as-usual.

**Table 2:** Outcome measures at baseline and follow-up presented as mean (SD).

	Baseline		Follow-up		Group × time interaction
	MORE (n = 37)	TAU (n = 34)	MORE (n = 18)	TAU (n = 25)	
ODI	19.70 (±7.66)	21.82 (±10.28)	20.83 (±8.38)	17.16 (±10.72)	*P* = 0.090
Depression and QoL					
MDI	25.16 (±13.62)	27.30 (±15.81)	23.44 (±13.78)	22.54 (±13.46)	*P* = 0.260
SF-12					
PCS	34.38 (±9.49)	34.73 (±9.57)	36.87 (±10.35)	39.25 (±11.53)	*P* = 0.990
MCS	40.32 (±11.31)	40.67 (±11.68)	39.88 (±11.60)	42.10 (±12.13)	*P* = 0.890
Mindfulness					
MRPS	19.11 (±9.55)	16.91 (±11.86)	31.11 (±4.28)	21.92 (±14.15)	*P* = 0.029
FFMQ-Acting with Awareness	24.86 (±6.48)	24.76 (±7.96)	26.67 (±6.32)	26.29 (±7.28)	
FFMQ-Describing	26.68 (±6.28)	27.70 (±7.21)	29.89 (±5.04)	28.83 (±8.72)	
FFMQ-Nonjudging of Inner Experience	25.65 (±6.34)	27.15 (±7.90)	27.78 (±6.75)	28.96 (±7.04)	
FFMQ-Nonreactivity to Inner Experience	20.95 (±5.30)	20.55 (±6.16)	22.00 (±2.89)	21.67 (±6.45)	
FFMQ-Observing	29.11 (±5.97)	27.00 (±7.81)	31.61 (±3.81)	27.37 (±7.82)	
FFMQ-Total	127.24 (±23.59)	127.15 (±25.94)	137.94 (±17.20)	133.13 (±28.34)	*P* = 0.035

FFMQ, Five-Facet Mindfulness Questionnaire; MCS, mental component score; MDI, Major Depressive Inventory; MRPS, Mindful Reappraisal of Pain Scale; ODI, Oswestry Disability Index; PCS, physical component score; SF-12, Short-Form Quality of Life Questionnaire.

**Table 3:** Baseline characteristics and outcome measures scores for completers vs noncompleters.

	Completers (n = 43)	Noncompleters (n = 28)	
Days from baseline visit to intervention start	41.86 (±4.28)	51.79 (±5.04)	*P* = 0.139
Demographic variables			
Age	45.21 (±10.74)	49.36 (±12.58)	*P* = 0.142
Condition duration	13.00 (±9.90)	13.65 (±12.84)	*P* = 0.812
Sex			*P* = 0.562*
Male	14 (33%)	11 (39%)	
Female	29 (67%)	17 (61%)	
No. of surgeries			*P* = 0.157*
1 surgery	7	2	
2 surgeries	0	1	
3 or more surgeries	2	0	
Race			
Anglo-American	35	23	*P* = 0.219*
Black	1	1	
Asian	2	—	
Hispanic/Latino	—	1	
Middle Eastern	1	1	
More than 1 race	3	2	
Other	1	—	
Disability and pain			
ODI	21.19 (±9.23)	20.00 (±8.77)	*P* = 0.591
PDQ			*P* = 0.676
>18	26	14	
12 and <19	8	7	
≤12	9	7	
Depression and QoL			
MDI	25.81 (±13.97)	26.74 (±15.86)	*P* = 0.798
SF-12			
PCS	34.30 (±10.11)	34.96 (±8.52)	*P* = 0.785
MCS	40.98 (±10.37)	39.75 (±13.02)	*P* = 0.673
Mindfulness			
MRPS	17.21 (±10.46)	19.36 (±11.12)	*P* = 0.412
FFMQ-Acting with Awareness	25.21 (±7.04)	24.19 (±7.45)	*P* = 0.564
FFMQ-Describing	27.21 (±6.90)	27.07 (±6.50)	*P* = 0.935
FFMQ-Nonjudging of Inner Experience	26.58 (±6.81)	26.00 (±7.67)	*P* = 0.742
FFMQ-Nonreactivity to Inner Experience	20.53 (±5.23)	21.11 (±6.42)	*P* = 0.683
FFMQ-Observing	27.44 (±7.02)	29.19 (±6.77)	*P* = 0.309
FFMQ-Total	126.98 (±22.45)	127.56 (±28.01)	*P* = 0.928

* *P*-value calculated using χ^2^.

FFMQ, Five-Facet Mindfulness Questionnaire; MCS, mental component score; MDI, Major Depressive Inventory; MRPS, Mindful Reappraisal of Pain Scale; ODI, Oswestry Disability Index; PCS, physical component score; PDQ, painDETECT Questionnaire; SF-12, Short-Form Quality of Life Questionnaire.

### 3.1. Intention-to-treat analyses

Because no potential covariates met the prespecified criterion of correlation *r* ≥ 0.3 with any outcome measure, models described below are unadjusted. The group × time interaction was the primary fixed effect of interest. Regarding ODI scores, the main effect of time was significant, *F*(1,46.77) = 7.54, *P* = 0.009. The group × time interaction was nonsignificant *F*(1,46.73) = 3.00, *P* = 0.09, indicating that change in ODI scores did not differ significantly between MORE and TAU groups.

Regarding daily pain intensity, the main effect of time was significant, *B* = −0.006 (SE = 0.002), *P* = 0.002. Importantly, the group × time interaction was significant, group × time *B* = −0.007 (SE = 0.003), *P* = 0.039, such that compared with TAU, participants in MORE reported significantly greater decreases in daily pain VAS over 8 weeks (14.0% decrease in pain intensity in MORE compared with a 6.8% decrease in TAU).

Regarding MDI scores, the main effect of time was significant, *F*(1,43.35) = 5.23, *P* = 0.027, with MDI scores improving across both groups. The group × time interaction was nonsignificant *F*(1,43.45) = 1.28, *P* = 0.26, indicating that change in MDI scores did not differ significantly between MORE and TAU groups.

Regarding SF-12 physical component scores (PCS), the main effect of time was significant, *F*(1,43.78) = 15.27, *P* < 0.001, with SF-12 PCS scores improving across both groups. The group × time interaction was nonsignificant *F*(1,43.78) = 0.00, *P* = 0.99, indicating that change in SF-12 PCS scores did not differ significantly between MORE and TAU groups. Regarding SF-12 mental component scores (MCS), the main effect of time was nonsignificant, *F*(1, 42.72) = 0.091, *P* = 0.764. The group × time interaction was nonsignificant *F*(1, 42.72) = 0.019, *P* = 0.890, indicating that change in SF-12 MCS scores did not differ significantly between MORE and TAU groups.

Regarding FFMQ-Total scores, the main effect of time was significant, *F*(1,44.13) = 11.90, *P* = 0.001, with FFMQ scores improving across both groups. Importantly, the group × time interaction was significant *F*(1,44.13) = 4.72, *P* = 0.035, such that patients in MORE evidenced significantly greater increases in FFMQ-total scores over time.

Regarding MRPS scores, the main effect of time was significant, *F*(1,49.18) = 44.32, *P* < 0.001, with MRPS scores improving across both groups. Importantly, the group × time interaction was significant *F*(1,49.18) = 5.03, *P* = 0.029, such that patients in MORE evidenced significantly greater increases in MRPS scores over time.

Of the 19 MORE group participants who completed follow-up surveys, 14 received the minimal intervention dose, ≥4 sessions.^26^ Because only 5 completers did not receive the minimal intervention dose, planned per-protocol models to assess sensitivity to treatment completion, as described in the study protocol, were omitted.

### 3.2. Adverse events

Throughout the yearlong data collection phase of the study, only one adverse event occurred related to physical health (unanticipated) and one related to mental/emotional well-being (anticipated) that were deemed likely to be associated with study procedures. The physical health adverse event was related to a previous condition that was addressed outside of the research setting. The other event was a result of emotional triggering in the process of discussions around physical pain during a weekly session; this participant chose to withdraw from the study. Neither of these adverse events occurred as a result of protocol deviations.

## 4. Discussion

### 4.1. Main findings

In this trial, both groups (MORE and TAU) reported positive changes in scores on the PDQ, MDI, SF-12 PCS, MRPS, and FFMQ. Only the measures of mindfulness, the MRPS and FFMQ, as well as the daily pain VAS, revealed significant group × time interactions, indicating superior improvement in MORE as compared to TAU. Our results for VAS represent a highly valuable finding, given that the implementation of the VAS, in our study, was similar to ecological momentary assessments—a rising gold standard for the assessment and collection of data on chronic pain.^54,68,69^ Despite other encouraging results, no positive findings were revealed for the impact of MORE on ODI scores, and in fact, the TAU group showed nonsignificant improvement in ODI scores, whereas the MORE group did not. Overall, our findings support a previous meta-analysis conducted on MORE, indicating its positive impact on pain intensity. We did not, however, confirm previously described reductions in depression symptoms within our data set.^61^

As we hypothesized daily pain intensity and predisability/postdisability scores would move together or not at all, it was to our surprise that the MORE group experienced decreases in daily pain intensity without decreases in disability. We suspect that the changes in pain intensity and disability were not married because of the extended condition duration for most of the patients enrolled in our sample (*x̄* = 13.26 years). It is well documented that patients with chronic pain experience high levels of pain interference,^44^ pain catastrophizing,^67^ and fear avoidance behaviors,^41^ all made worse by the social isolation frequently occurring during the COVID-19 pandemic.^6,11,36^ In addition, the ODI assesses disability through questions regarding activities of daily living. As people's activities of daily living changed drastically throughout pandemic-related lockdowns, the ODI may not have captured elements of disease severity that were modified with MORE.

### 4.2. Limitations, strengths, and recommendations

The strengths of this study remain as we proposed in the study protocol. First, previous MBIs evaluating patients with cLBP have conducted stratified analyses of patients with radicular symptoms; to date, this is the only randomized controlled trial evaluating an MBI for patients with LR specifically. Next, this trial used a relatively novel MBI, MORE, which is specific to pain conditions. In addition, with the ongoing COVID-19 pandemic, which created significant obstacles in the process of recruitment and participant retention, the virtual delivery of the intervention made this program accessible during pandemic-related lockdowns, for patients with chronic pain lacking mobility, transportation services, and geographic accessibility. Next, at the time of protocol development and publication, the MRPS was only an experimental tool and had not yet been validated or used in published clinical trials. Our trial was able to use the MRPS as a validated measure of pain reappraisal and mindful coping. Finally, previous research has found that those reporting a higher degree of social isolation report increased pain interference,^44^ and this trial was able to successfully recruit participants and produce positive clinical outcomes while navigating restrictions on research activities because of pandemic-related lockdowns.

This study faced significant challenges in its ability to successfully retain participants, likely because of the ongoing COVID-19 pandemic at the start of enrollment and throughout delivery of the intervention. Although dropout and lost to follow-up were greater than anticipated, this was recognized during data collection, and recruitment timing was adjusted accordingly to increase the sample size. Many clinical trials of this scale compensate participants directly for their time as an incentive for continued study participation.^2,62^ In this trial, we were unable to compensate participants for their involvement, which may have acted to decrease retention. In addition, because of limited resources, we were forced to recruit participants into fewer and larger groups above previous recommendations for psychotherapy interventions.^15^ The size of the groups may have in itself negatively affected the therapeutic benefit that participants were receiving by reducing their time and attention from the MORE instructor while attending weekly sessions. Although virtually delivered MBIs have been shown to be efficacious,^10,21,30,33,38,43,55,73,74^ this study encountered challenges with the virtual delivery of MORE. If we are to recognize the therapeutic effect of groups^52^ and the microinteractions that make those groups into successful communities, it is important to recognize that virtual psychotherapy programs lack the opportunity for participants to connect when not directly engaged in group discussion and teaching by the facilitator. Small interactions had before and after sessions, during restroom breaks, and between weekly meetings create a sense of community that decreases the impact of social isolation on factors such as pain interference.

Finally, 2 protocol modifications were made during study execution: one in data collection and one in statistical analysis. First, due to staff limitations, it was required that the study coordinator run a small number of follow-up study visits. This team member was unblinded to participant group assignment because they had conducted the randomization sequence and group assignment and acted as a back-up MORE instructor throughout the program. Second, because so few participants had low study attendance, per-protocol and sensitivity analysis was not performed.

Future studies implementing web-based mindfulness interventions should consider creative approaches to the development of virtual communities, such as technology information sessions, an in-person meet-and-greet before or during the intervention, ice-breakers or get-to-know-you exercises before beginning psychoeducational content in weekly sessions, etc. In addition, the MORE instructor reported discussing with many participants the positive impact that MORE was having on their mental health and general sense of quality of life, from many participants unable to attend weekly sessions. This reporting was counter to other participants who seemed to attend out of a sense of obligation to finish the trial rather than because they were benefiting from the practice. As psychotherapy programs, and active interventions generally, are highly dependent on the participants' level of effort and interest, future research should attempt to determine participants' motivational orientation towards the practice before and after intervention and consider stratification by motivational group in statistical analyses. This determination can be accomplished using existing models of motivation and behavior change such as the transtheoretical model of change,^64^ self-determination theory,^18^ self-efficacy theory,^7^ or a combination. Correctly identifying an individual's motivational orientation is likely best accomplished through a combination of quantitative and qualitative data collection using self-report questionnaires and phenomenologic interviews. Future studies' outcomes may also benefit from taking place in a postpandemic environment that is less restricted than during the trial period. This may have a meaningful impact on the efficacy of MORE in similar trials.

In addition, it is important that as efficacy is established for MBIs across more conditions types, research moves toward pragmatic and multimodal trial designs that are highly representative of target subpopulations and considerate of real-world obstacles to care for those respective patients.^45,49,63,71^ In this subpopulation, we hypothesize that patients with LR would have highly benefited from the implementation of a multimodal mindfulness and movement program that could have helped mitigate the development of or reduced existing fear avoidance behaviors and nociplastic pain.

## 5. Conclusion

Virtual delivery of MORE significantly reduced daily pain intensity, but not disability or depression symptoms, in patients with LR as compared to TAU. This is possibly a result of fear avoidance behaviors because of patients in our sample having lived with their conditions for an average of ∼13 years. Undergoing training in MORE also significantly increased trait mindfulness and mindful reappraisal of pain. Future trials should attempt to replicate the observed effect of MORE on daily pain intensity and consider the use of multimodal interventions, such as movement programs, to enhance the effect of MORE on disability in patients with LR.

## Disclosures

The authors have no conflict of interest to declare.
